# Transplantation of SDF-1α-loaded liver extracellular matrix repopulated with autologous cells attenuated liver fibrosis in a rat model

**DOI:** 10.17179/excli2022-4761

**Published:** 2022-04-22

**Authors:** Mostafa Najar-Asl, Hossein Bahadoran, Mohammad-Hossein Asadi, Mona Saheli, Mohammad-Hassan Asghari, Niloofar Sodeifi, Mohammad Kazemi Ashtiani, Massoud Vosough, Hossein Baharvand, Abbas Piryaei

**Affiliations:** 1 Department of Stem Cells and Developmental Biology, Cell Science Research Center, Royan Institute for Stem Cell Biology and Technology, ACECR, Tehran, Iran; 2 Department of Anatomical Sciences, School of Medical Sciences, Baqiyatallah University, Tehran, Iran; 3 Department of Anatomical Sciences, Afzalipour Faculty of Medicine, Kerman University of Medical Sciences, Kerman, Iran; 4 Animal Core Facility, Reproductive Biomedicine Research Center, Royan Institute for Biotechnology, ACECR, Tehran, Iran; 5 Department of Andrology, Reproductive Biomedicine Research Center, Royan Institute for Reproductive Biomedicine, ACECR, Tehran, Iran; 6 Department of Cell Engineering, Cell Science Research Center, Royan Institute for Stem Cell Biology and Technology, ACECR, Tehran, Iran; 7 Department of Regenerative Medicine, Cell Science Research Center, Royan Institute for Stem Cell Biology and Technology, ACECR, Tehran, Iran; 8 Department of Developmental Biology, School of Basic Sciences and Advanced Technologies in Biology, University of Science and Culture, Tehran, Iran; 9 Department of Biology and Anatomical Sciences, School of Medicine, Shahid Beheshti University of Medical Sciences, Tehran, Iran; 10 Department of Tissue Engineering and Applied Cell Sciences, School of Advanced Technology in Medicine, Shahid Beheshti University of Medical Sciences, Tehran, Iran

**Keywords:** liver extracellular matrix scaffold, stromal derived factor-1alpha, granulocyte colony stimulating factor, endogenous progenitor cells mobilization, in vivo tissue engineering, liver fibrosis

## Abstract

Cell-based therapy and tissue engineering are promising substitutes for liver transplantation to cure end-stage liver disorders. However, the limited sources for healthy and functional cells and poor engraftment rate are main challenges to the cell-based therapy approach. On the other hand, feasibility of production and size of bioengineered tissues are primary bottlenecks in tissue engineering. Here, we induce regeneration in a rat fibrotic liver model by transplanting a natural bioengineered scaffold with a native microenvironment repopulated with autologous stem/progenitor cells. In the main experimental group, a 1 mm^3^ stromal derived factor-1α (SDF-1α; S) loaded scaffold from decellularized liver extracellular matrix (LEM) was transplanted (Tx) into a fibrotic liver and the endogenous stem/progenitor cells were mobilized via granulocyte colony stimulating factor (G-CSF; G) therapy. Four weeks after transplantation, changes in liver fibrosis and necrosis, efficacy of cell engraftment and differentiation, vasculogenesis, and liver function recovery were assessed in this (LEM-TxSG) group and compared to the other groups. We found significant reduction in liver fibrosis stage in the LEM-TxSG, LEM-TxS and LEM-TxG groups compared to the control (fibrotic) group. Liver necrosis grade, and alanine transaminase (ALT) and aspartate transaminase (AST) levels dramatically reduced in all experimental groups compared to the control group. However, the number of engrafted cells into the transplanted scaffold and ratio of albumin (Alb) positive cells per total incorporated cells were considerably higher in the LEM-TxSG group compared to the LEM-Tx, LEM-TxS and LEM-TxG groups. Serum Alb levels increased in the LEM-Tx, LEM-TxS, and LEM-TxG groups, and was highest in the LEM-TxSG group, which was significantly more than the fibrotic group. Small vessel formation in the LEM-TxSG group was significantly higher than the LEM-Tx and LEM-TxS groups. Totally, these findings support application of the *in vivo* tissue engineering approach as a possible novel therapeutic strategy for liver fibrosis.

## Introduction

The liver is a vital and complicated organ that contributes to most biochemical processes of metabolism, homeostasis, and detoxification (Ma et al., 2016[24]; Zhang et al., 2018[54]). Unfortunately, because of sedentary lifestyles, deaths caused by liver diseases are increasing worldwide (Mazza et al., 2015[29]). While liver transplantation is the gold standard treatment for end-stage liver diseases, this therapy is limited because of the shortage of suitable donors, intensive surgery and possibility of transplanted organ rejection (Nobakht Lahrood et al., 2020[34]; Lotfinia et al., 2016[23]). Furthermore, the main restriction of cell therapy approaches, as an alternative treatment, is poor cell engraftment, especially in the cases of liver fibrosis and cirrhosis, which are the consequences of all chronic liver diseases (Lee et al., 2016[18]; Yang et al., 2018[52]; Oldhafer et al., 2016[35]). Liver fibrosis leads to progressive replacement of functional liver tissue with collagen I/III-rich extracellular matrix (ECM) (Shiha et al., 2020[44]). In this situation, the fibrotic liver tissue disrupts both hepatic specific functions and the normal architecture, which precludes their competency for cell homing. Although some studies have shown the effective role of cells or stem cells' therapy for the treatment of liver fibrosis, this approach remains constricted by major issues such as the number of cells, route of cell transplantation, low cell proliferation and survival rate, and ectopic cell homing and unexpected differentiation that may lead to oncogenicity of the transplanted cells (Forbes et al., 2015[10]; Eom et al., 2015[9]; Shiha et al., 2020[44]). 

Besides the cell therapy approach, bioengineering strategies have emerged that deal with developing transplantable bioengineered liver tissues as a promising alternative *in vivo* treatment or for long-term hepatic functional restoration *in vitro*. Successful tissue engineering depends on synergy of a suitable three-dimensional (3D) scaffold to support contributing cell engraftment and angiogenesis, as well as active biomolecules and a dynamic microenvironment, which are the major parameters that lead to subsequent functionality of the developed construct (Zhang et al., 2018[54]; Lee et al., 2017[17]). 

Because of the presence of tissue-specific components, the liver extracellular matrix (LEM) can provide an ultimate suitable microenvironment for liver tissue engineering in terms of cell attachment, differentiation, proliferation, and function (Sellaro et al., 2010[43]). A LEM-derived scaffold obtained through decellularization method, by retaining native tissue organization and vascular microarchitecture could provide a perfusion system for better survival and maintenance of engrafted cells (Zhang et al., 2009[55]). Current studies have assessed decellularization approaches that preserved the microstructure, vascular pattern, and biochemical and biophysical characteristics of native liver ECM, which indicates significant advancements in tissue engineering (Uygun et al., 2010[47]; Damania et al., 2018[5]). 

Achievement of *in vitro* tissue engineering depends on the availability of suitable cell sources, strategies used for cell isolation and expansion, and cell penetration into a 3D scaffold (Sauer et al., 2014[42]), all of which are main limiting factors for this approach. Accordingly, promising attempts have been made for alternative *in vivo* tissue engineering, in which a single component of traditional triad for tissue engineering or their combination is directly implanted in the body to provide support for neotissue formation (McCullen et al., 2011[30]). This strategy may have distinct advantages over the *in vitro* tissue engineering, mainly through the recruitment of endogenous cells. Because of a natural bioreactor provided by the circulatory system (Martinod et al., 2017[26]; Jana et al., 2019[15]), in addition to local angiogenesis that provides necessary nutrient and environmental factors, the continuous endogenous cell mobilization improves homing, survival, proliferation, and differentiation at the site of the regenerating tissue (Leyendecker et al., 2018[19]; Mazza et al., 2018[28]).

Many studies reported the participation of endogenous hematopoietic and mesenchymal stem cells in liver regeneration after a liver injury (Ahmadi et al., 2018[1]; Wesson and Cameron, 2011[51]; Zhai et al., 2018[53]). Exogenous stem cells would decrease both the safety and efficiency of tissue regeneration because of immune system activation and graft rejection. Also, isolation and preparation of autologous stem cells is time consuming and *in vitro* maintenance of the functionality and quantity of these cells remains to be determined (Ahmadi et al., 2018[1]; Zhai et al., 2018[53]). Therefore, mobilization of endogenous stem and progenitor cells has gained momentum for *in vivo* tissue engineering approaches (Hu et al., 2019[14]).

In the *in vitro* tissue engineering approaches, selection of an appropriate cell source, an appropriate method for the cell and scaffold combination, the size of the engineered construct, induction of angiogenesis, and possibility of necrotic center formation are other main challenges (Park et al., 2016[36]).

Attempts at the *in vivo* tissue engineering approach not only recruit endogenous cells but also provide circulating growth factors and cytokines, and stimulate angiogenesis in the created tissue. Consequently, under such conditions, an efficient supply of oxygen and nutrients in the constructing microenvironment can lead to long-term cell survival and keep the tissue-specific functions of the contributing cells (Vishwakarma et al., 2019[48]). 

Hence, the *in vivo* development of biomimetic engineered liver tissue constructs by endogenous cells and suitable native scaffold transplantation allows us to overcome difficulties such as technical handling of the graft and prevention of necrotic areas through neoangiogenesis and connection of new vessels with the host vascular system (Tadakuma et al., 2013[46]). 

The mechanisms of hepatic regeneration after a liver injury are well-understood. The proliferation of mature hepatocytes and local stem cells promptly regenerates the liver mass and function in sub-lethal settings. When this mechanism is blocked or is insufficient, mobilized bone marrow-derived stem cells (BMSCs) participate in regeneration of the injured liver. Pharmacological mobilization of BMSCs has emerged as the standard of care for patients with liver failure who require liver transplantation (Ahmadi et al., 2018[1]; Danilova et al., 2019[6]). 

Current investigations have found many factors involved in BMSC mobilization (Fu et al., 2019[12]). Granulocyte colony stimulating factor (G-CSF) and stromal derived factor-1 (SDF-1)/CXC chemokine receptor 4 (CXCR4) axis play critical roles in BMSC mobilization, chemo-attraction, and homing of cells to the site of the injury. Some studies demonstrated that the combination of such factors could promote liver regeneration (Zhai et al., 2018[53]). Therefore, optimization of autogenous BMSCs mobilization could be effectively utilized to treat patients with end-stage liver failure (Ahmadi et al., 2018[1]; Fu et al., 2019[12]). 

The aim of this study was to induce regeneration in rat fibrotic liver tissue through an *in vivo* tissue engineering approach. For this purpose, we transplanted a xenograft of a SDF-1α loaded mouse liver ECM, as a chemoattractant scaffold for cell penetration, and mobilized endogenous cells, via G-CSF therapy in an attempt to improve cell engraftment in the scaffold. The results provided a proof-of-concept for regeneration of fibrotic liver using cell-free liver ECM scaffolds and induction of G-CSF/SDF-1α axis for endogenous cell recruitment.

## Materials and Methods

The current study was approved by the Ethical Review Board at Royan Institute for Stem Cell Biology and Technology, Tehran, Iran (code no: IR.ACECR.ROYAN.REC.1395.182). All materials were purchased from Sigma-Aldrich (St. Louis, MO, USA) unless otherwise stated.

### Preparation of liver extracellular matrix scaffold

**Figure 1:**
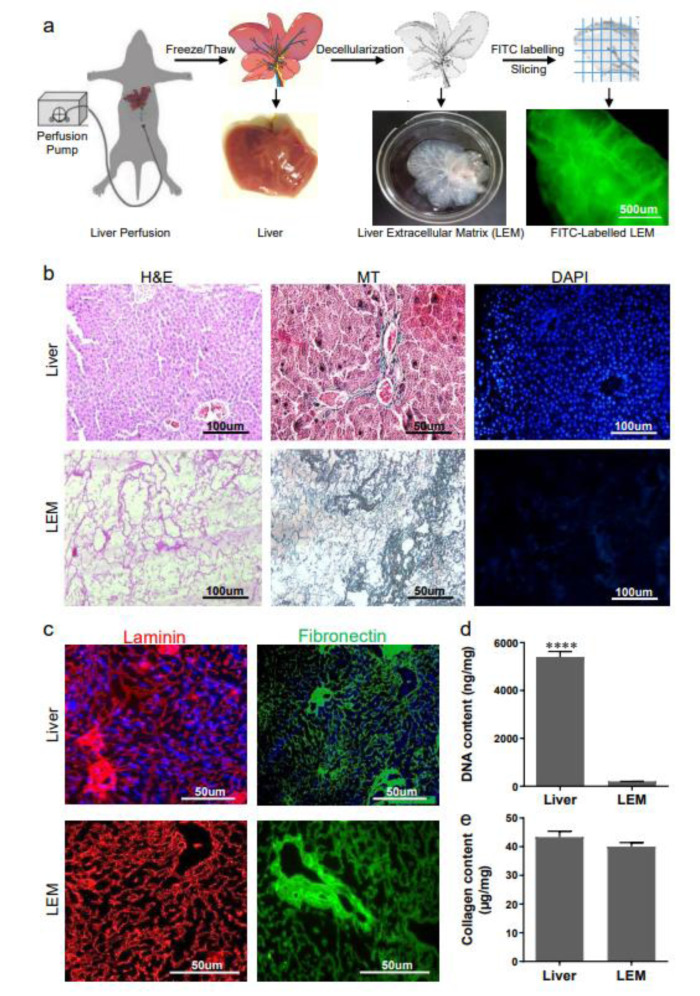
Liver-derived extracellular matrix preparation and characterization. a) Representative scheme for the main steps of liver perfusion, cryochemical decellularization, and labeling. For this purpose, the portal vein was cannulated and perfused with PBS, then the liver was frozen, thawed and perfused with Triton-X100 and Ammonium hydroxide until the liver became clear and transparent. The liver extracellular matrix (LEM) was sliced and labeled with fluorescein isothiocyanate (FITC), to be detectable in the transplantation site. b) Micrographs of liver and LEM sections stained with hematoxylin and eosin (H&E, to show the presence or absence of native cells), 4',6-diamidino-2-phenylindole (DAPI, to demonstrate nuclei elimination), or stained with Masson's trichrome (MT, to show collagen preservation after decellularization). c) Immunofluorescent micrographs of liver sections and LEM to visualize the retention of laminin and fibronectin in the LEM. As can be seen, both important glycoproteins retained in the resultant LEM as well as native liver tissue. d and e) DNA and collagen content quantification assays, as two important parameters for quality of resultant ECM, confirmed efficient DNA removal and preservation of collagen content during the decellularization process. ****P<0.0001

Liver decellularization was performed on a male healthy adult NMRI mouse using the cryochemical method, according to Jiang et al. (2014[16]) with minor modifications. After deep anesthesia, the portal vein was cannulated with a 22-G needle and perfused with PBS for 10 min, then the liver was frozen at 80 °C for 24 h. Next, the frozen liver was thawed and perfused with 2.5 % Triton-X100 and 0.25 % NH_4_OH until the liver became clear and transparent. The LEM was sliced and labeled with fluorescein isothiocyanate (FITC). Briefly, the LEM was immersed in a solution of 0.1 M sodium carbonate buffer (pH 9) under continuous shaking, followed by treatment with ammonium chloride (50 mM) and glycerol (5 % v/v). Finally, the FITC-labeled LEM were cut into 1×1 mm pieces and stored at -20 °C until transplantation (Figure 1a(Fig. 1)).

### Characterization of the liver extracellular matrix 

#### Histological analysis

For histological characterization, 6 µm thick sections were prepared from the LEM and intact liver after formalin fixation and paraffin embedding. The sections were stained using hematoxylin and eosin (H&E) or 4',6-diamidino-2-phenylindole (DAPI; D8417) to evaluate the presence of cellular components. Masson's trichrome (MT) staining was performed to visualize the presence of collagen. 

#### Preservation of laminin and fibronectin assessments

In order to confirm preservation of the main ECM glycoproteins in the LEM, the tissue sections were immunofluorescently stained using primary antibodies against laminin (ab30320, Abcam) and fibronectin (ab2413, Abcam). Then, the sections were incubated with Alexa Fluor 568- or 488-conjugated secondary antibodies (A100-40, Invitrogen and ab97199, Abcam, respectively). The nuclei were counterstained with DAPI. The stained sections were analyzed using a fluorescent microscope (BX51; Olympus, Japan).

#### DNA content analysis 

Quantification of DNA content in the LEM and intact liver was performed as previously described (Saheli et al., 2018[39]; Nobakht Lahrood et al., 2020[34]) using the phenol-chloroform assay. Briefly, the samples were cut into small pieces and digested with proteinase K (03115836001; Roche, Mannheim, Germany) at 56 °C. Centrifugation was performed and the supernatant was collected. Then, the DNA fragments were sedimented, washed with cold ethanol, and dried. Finally, the DNA fragments were dissolved in distilled water and the absorbance was quantified at 260 nm using a spectrophotometer (WPA Biowave II, Cambridge, UK).

#### Collagen content assessment

The collagen content in the resultant LEM and intact liver was measured according to Namiri et al. (2018[32]) using colorimetric Blyscan assay kits (Biocolor, Carrickfergus, UK). Briefly, the samples were digested with 0.1 M HCl‐pepsin and incubated in a solution that included Sirius red dye. Collagen content was defined by measuring the absorbance at 555 nm (WPA Biowave II, Cambridge, UK).

### Stromal derived factor-1α immobilization to the liver extracellular matrix

After LEM characterization, mouse recombinant SDF‐1α (PeproTech) was loaded into the scaffold according to Namiri et al. (2018[32]) using an m‐maleimidobenzoyl‐N‐hydroxysulfosuccinimide ester (sulfo-MBS). For this purpose, the LEM slices were immersed in 1 mg/ml sulfo‐MBS in PBS for 1 h, then incubated in a solution of 500 ng/ml SDF‐1α in PBS for 4 h. Free SDF‐1α was removed by washing three times with PBS. Finally, the amount of immobilized SDF‐1α was measured by quantification of remaining growth factor in the washing solutions by using an SDF‐1α sandwich enzyme‐linked immunosorbent assay (ELISA, MCX120 R&D Systems) kit.

### Animal model of liver fibrosis and experimental groups 

**Figure 2:**
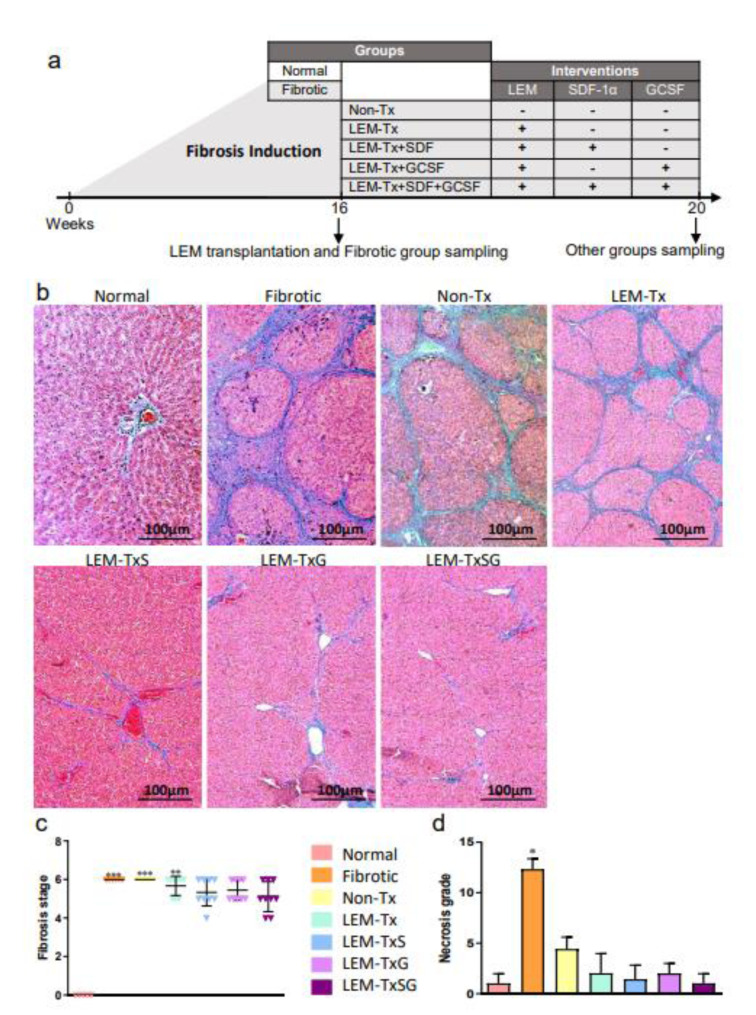
The impact of the treatment regimens on histopathologic characteristics of the liver. a) The scheme simply shows the experimental groups and their interventions as well as sampling time points. Normal group: healthy rats; Fibrotic group: the rats which were sacrificed after 48 times of TAA administration at the end of 16^th^ week; Non-transplanted (Non-Tx) group: the rats which were sacrificed 4 weeks after the last injection of TAA; LEM-transplanted (LEM-Tx) group, received the LEM transplantation at the end of the 16^th^ week and were sacrificed at the 20^th^ week; LEM-TxS group: which was the same as the former group but received a transplantation of SDF-1α loaded LEM; LEM-TxG group: which was the same as the LEM-Tx group, but they received an IP injection of 100 µg/kg G-CSF after LEM transplantation; LEM-TxSG group: which received a transplantation of SDF-1α loaded LEM and treatment with G-CSF. b) Masson's trichrome (MT) staining of liver sections representatively demonstrate thick periportal fibrotic tissue in the fibrotic, Non-Tx and LEM-Tx groups, and some fibrolysis in the LEM-TxS, LEM-TxG and LEM-TxSG groups. c) Liver fibrosis stage quantification based on Rosai scoring (2011) confirms significant elevations (**P<0.01 and ***P<0.001) in the fibrotic, Non-Tx and LEM-Tx groups compared to the normal group. While there were no significant differences in the fibrosis stages of the LEM-TxS, LEM-TxG and LEM-TxSG groups in comparison to the normal group. d) Quantification of necrosis grade showed a significant reduction in all of the groups compared to the fibrotic group. *P<0.05 versus the other groups

Liver fibrosis was induced in male Wistar rats (6 weeks old) according to Wallace et al. (2015[49]), using an intraperitoneal (IP) injection of thioacetamide (TAA) dissolved in normal saline that was administered at a dose of 240 mg/kg, three times per week for 16 weeks. The healthy rats (n=5) each received an IP injection of normal saline (normal group). The fibrotic liver model rats were randomly divided into six experimental groups (n≥5 per group, Figure 2a(Fig. 2)), as follows: i) fibrotic group, which were sacrificed at the end of TAA administration at the 16^th^ week; ii) non-transplanted (Non-Tx) group, which were sacrificed four weeks after the last injection of TAA (week 20); iii) LEM-transplanted (LEM-Tx) group, received the LEM transplantation at the end of the 16^th^ week and were sacrificed at the 20^th^ week; iv) LEM-TxS group, which was the same as the former group but received a transplantation of SDF-1α loaded LEM; v) LEM-TxG group, which was the same as the LEM-Tx group, but they received an IP injection of 100 µg/kg G-CSF after LEM transplantation; and vi) LEM-TxSG group, which received a transplantation of SDF-1α loaded LEM and treatment with G-CSF. At the appropriate times (at the end of the 16^th^ or 20^th^ week), the rats were sacrificed by cervical dislocation, and their blood and liver tissue samples were harvested for further evaluations.

### Surgical procedure and liver extracellular matrix transplantation

LEM transplantation was performed according a method created by our laboratory at Royan Institute, Tehran, Iran. Briefly, the mice were anesthetized by inhalation of isoflurane and placed on a warm stage (37 °C), then the abdominal skin was shaved and disinfected by 70 % ethanol. After opening the abdominal wall, an incision was made in the middle lobe of the liver with a number 11 surgical razor. Finally, a 1 mm^3^ piece of LEM was placed in the incision area and the incision was covered with biological glue to prevent bleeding.

### Evaluation of the treatment regimens in the fibrotic liver

The animals were sacrificed four weeks after the last TAA injection or transplantations and corresponding samples were collected to investigate the impact of the treatment regimens on the fibrotic liver model. We were particularly interested in the main experimental (LEM-TxSG) group, which received a transplant of SDF-1α loaded LEM in combination with G-CSF therapy. 

#### Histopathological examinations

For histopathological examinations, the liver tissues that contained the transplanted LEM were fixed and processed. After paraffin embedding and sectioning, H&E and MT staining were performed to quantify the fibrosis stage and necrosis grade. The fibrosis stage and necrosis grades (piecemeal necrosis, confluent necrosis, focal lytic necrosis, apoptosis, focal inflammation, and portal inflammation) in the liver sections were evaluated according to a previously described method (Rosai, 2011[38]) (Supplementary information). Two pathologists, blinded to the groups, independently assessed and scored the fibrosis levels in the slides.

#### Engrafted cells in the transplanted liver extracellular matrix and their fate

In order to demonstrate the engrafted cells and their fate in the transplanted LEM, at four weeks following transplantation, the liver tissue sections from the transplanted areas were stained with DAPI and immunostained against albumin (Alb) using (A80-229A, Bethyl Laboratories) a primary antibody and Alexa Fluor® 568 conjugated (ab175474) secondary antibody. The 6-µm-thick sections were stained with DAPI to quantify the total engrafted cells in the transplanted LEM. After identifying the FITC-labeled transplanted LEM, 25 sections at an equal distance from each other were selected from each animal and all engrafted cells were counted using a fluorescent microscope with a 10× objective lens. Furthermore, the percent of Alb-positive cells per total engrafted cells in the transplanted LEM was calculated in the evaluated sections.

#### Angiogenesis in the transplanted liver extracellular matrix

We evaluated angiogenesis in the transplanted LEM by immunohistochemical analysis using anti-CD31, an endothelial cell marker, primary (ab28364) and HRP-conjugated secondary (65-6120, Invitrogen) antibodies. Finally, the nuclei were counterstained with hematoxylin. Micrographs for a total of 30 random fields per group were captured and analyzed independently to calculate the number of blood vessels. We demonstrated the criteria for small, medium, and large vessels by diameter according to Bukenya et al. (2020[3]), as follows: small (vessels without lumen), medium (vessels with lumen 2-10 µm in diameter), and large (vessels with lumen larger than 10 µm in diameter).

#### Liver functions

Serum samples were collected four weeks after transplantation for analysis of alanine transaminase (ALT), aspartate transaminase (AST), and Alb levels in the rats' sera by commercially available kits (20764949322, 20764957322, 400-0029, respectively, Roche).

### Statistical analysis

Data analysis was performed using GraphPad Prism 6.0. One-way repeated measures analysis of variance (ANOVA) followed by the Tukey post hoc test for multiple group comparison or Kruskal-Wallis were presented as both number and percentage values. The data are presented as mean ± SD (standard deviation). P<0.05 was considered to be statistically significant.

## Results

### Decellularization efficiency

After complete decellularization of the liver lobes, we observed that they became translucent due to the removal of the cells, and the vascular structure was intact and visible (Figure 1a(Fig. 1)). Cell removal was qualitatively confirmed by histological analysis with H&E and DAPI staining, while MT staining showed the collagen content in the resultant LEM (Figure 1b(Fig. 1)). Preservation of laminin and fibronectin, which were determined by immunofluorescence in the resultant LEM appeared to be the same as native liver tissue (Figure 1c(Fig. 1)). Quantitative assessments of DNA and collagen content revealed that the DNA content of the LEM was less than 3.7 % of the original liver. However, there were no significant differences between collagen content of the resultant LEM and native liver tissue (Figure 1d(Fig. 1)). 

### Validation of stromal derived factor-1α immobilization on the liver extracellular matrix

We measured the unreacted growth factor in the washing solution to validate SDF‐1α immobilization onto LEM after the loading process. The amount of immobilized SDF-1α was 32 ± 6 ng/mg of the LEM. 

### The impact of the treatment regimens to rescue fibrotic liver

#### Reduction of liver fibrosis and necrosis

Collagen deposition, as a fibrosis characteristic, was evaluated by MT staining. In the fibrotic group, we observed thick considerable collagenous septa in the periportal area of the liver lobules that also spread finely into the lobule parenchyma. This fibrotic condition was seen in the Non-Tx and LEM-Tx groups, while there was remarkable resolution in the fibrotic tissue in the LEM-TxS, LEM-TxG, and LEM-TxSG groups (Figure 2b(Fig. 2)). Quantification of the fibrosis stages showed no significant differences in the fibrosis stages of the LEM-TxS, LEM-TxG and LEM-TxSG groups compared to the normal group (Figure 2.c(Fig. 2)). Evaluation of necrosis grade showed a significant (P<0.05) increase in the fibrotic group in comparison to the other groups (Figure 2d(Fig. 2)). 

By taking into consideration these two parameters, it could be suggested that the LEM-TxS, LEM-TxG and LEM-TxSG treatments could considerably rescue the fibrotic liver in terms of fibrosis stage and necrosis grade. 

#### Cell engraftment, differentiation, and restoration of liver function 

**Figure 3:**
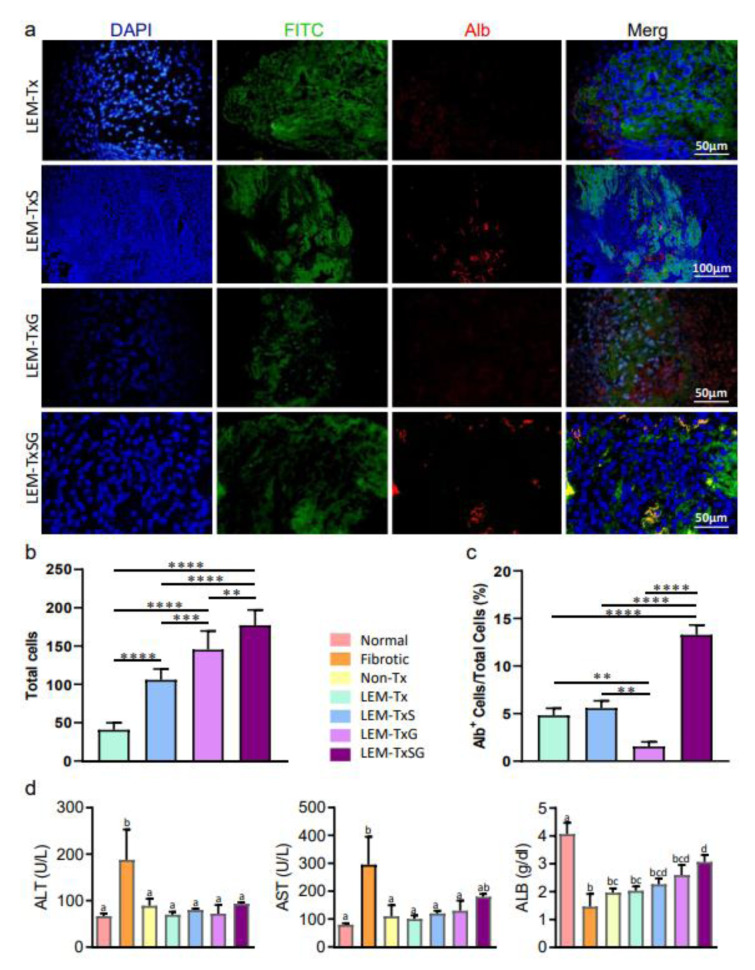
Endogenous cells engraftment and differentiation in the transplanted liver extracellular matrix to restore liver function. a) To demonstrate the engrafted cells and their fate in the transplanted LEM, four weeks after transplantation, the liver tissue sections from the transplanted areas were immunostained using antibody against Alb. 4',6-diamidino-2-phenylindole (DAPI) stained nuclei demonstrated engraftment of cells in the transplanted fluorescein isothiocyanate (FITC)-labeled LEM in all experimental groups which received LEM transplantation. b) Quantification of the findings demonstrated that the LEM-Tx group had the least cell engraftment, while there was significantly more cell engraftment in the LEM-TxS, LEM-TxG and LEM-TxSG groups, respectively compared to their former groups. c) Furthermore, Alb expression by the engrafted cells was considered to indicate cell differentiation toward the hepatocyte fate. The quantitative data indicated that the lowest and the highest percent of Alb-positive (Alb^+^) cells were in the LEM-TxG and LEM-TxSG groups, respectively. Taking together, comprehensive quantification analysis of total engrafted cells and the percentage of Alb^+^ cells demonstrate that transplantation of stromal derived factor-1α (SDF-1α) loaded LEM in combination with granulocyte colony stimulating factor (G-CSF) therapy provide a more suitable context for endogenous cell engraftment and differentiation to hepatocytes. **P<0.01, ***P<0.001, ****P<0.0001. d) Comparison of serum levels of biomarkers for liver injury (alanine transaminase [ALT] and aspartate transaminase [AST]) and functionality (Alb) demonstrated some enhanced effectiveness for the LEM-TxSG group. Non-identical letters show significant differences to each other, at least at *P*<0.05.

We evaluated the total engrafted cells in the transplanted LEM four weeks after transplantation in order to determine which treatment regimens were more suitable for cell recruitment in the fibrotic liver tissue (Figure 3a(Fig. 3)). Quantitative findings clarified that the LEM-Tx group had the least cell engraftment; SDF‐1α loading or G-CSF therapy significantly increased cell engraftment in the LEM-TxS and LEM-TxG groups. Finally, simultaneous application of SDF‐1α loaded LEM and G-CSF therapy significantly enhanced cell engraftment compared to each treatment individually (Figure 3b(Fig. 3)). 

Alb expression by the engrafted cells was considered to indicate cell differentiation toward the hepatocyte fate. The percentage of Alb expressing cells among the total engrafted cells was calculated. The results indicated that the lowest and the highest percent of Alb positive cells were in the LEM-TxG and LEM-TxSG groups, respectively (Figure 3c(Fig. 3)). 

The serum levels of some specific liver biomarkers were measured to investigate the impact of the differentiated cells on restoration of fibrotic liver function. As expected, the amount of ALT and AST, as liver injury markers, significantly increased in the fibrotic group compared to the normal group. However, the levels of these markers decreased to normal levels in all of the other experimental groups (Figure 3d(Fig. 3)). Liver dysfunction mainly causes a reduction in serum Alb levels, and our results showed a dramatic decrease in this marker in the fibrotic group compared to the normal group. However, serum Alb levels increased in the Non-Tx, LEM-Tx, LEM-TxS, LEM-TxG, and LEM-TxSG groups. The LEM-TxSG group had significantly higher Alb levels compared to the fibrotic, Non-Tx, and LEM-Tx groups (Figure 3d(Fig. 3)).

Taken together, not only did cell engraftment significantly improve in the LEM-TxSG group, but also the cell differentiation and hepatic functions considerably increased with simultaneous use of SDF‐1α loaded LEM and G-CSF therapy in the fibrotic livers.

#### Angiogenesis in the transplanted liver extracellular matrix 

**Figure 4:**
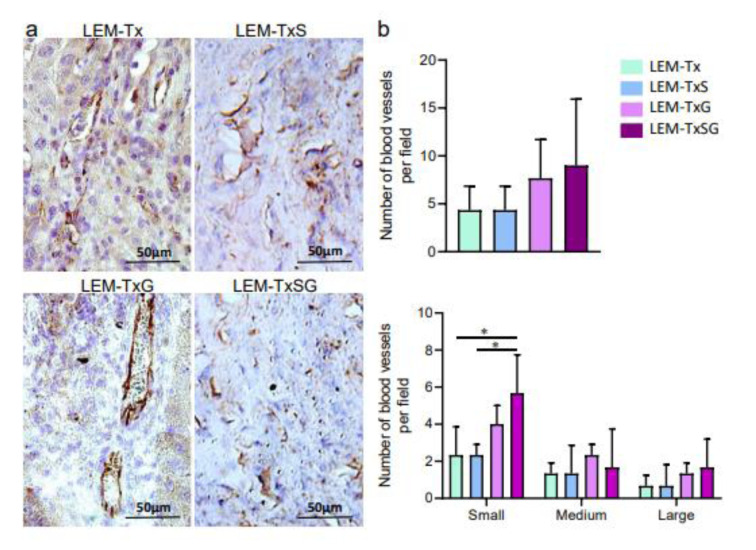
Angiogenesis in the transplanted liver extracellular matrix. a) Four weeks following transplantation, the liver tissue sections from the transplanted areas were immunostained against CD31 to demonstrate vascularization in the transplanted LEM. b) Quantitative analysis of the blood vessels shows that while there are no significant differences in the total vessels of all experimental groups which received LEM transplantation, the LEM-TxSG group has significantly more small vessels, which may be as representative for liver sinusoid, compared to the LEM-Tx and LEM-TxS groups. *P<0.05

Oxygen and metabolites are necessary for engrafted cells, as part of liver tissues; therefore, we evaluated new vessel formation in the transplanted LEM (Figure 4a(Fig. 4)). Although there was angiogenesis, we observed no significant difference between the numbers of total blood vessels in the different groups. However, when we separately considered the number of small, medium or large vessels, we found a significant increase in the number of small blood vessels in the LEM-TxSG group compared to the LEM-Tx and LEM-TxS groups (Figure 4b(Fig. 4)). These findings suggested that concurrent applications of SDF‐1α loaded LEM and G-CSF therapy might advance neovascularization concerning small blood vessels.

## Discussion

Because of the complications with liver transplantation, cell therapy is considered to be an alternative treatment method for patients with end-stage liver diseases. However, poor engraftment of transplanted cells is a major limitation of cell-based therapy methods (Zhao et al., 2019[56]; Wan et al., 2013[50]). Therefore, generating bioartificial liver constructs by using tissue engineering approaches is a goal of recent studies. 

Selection of the best cell for bioartificial liver generation is a challenge in tissue engineering approaches. Primary human hepatocyte isolation and expansion or the establishment of an efficient protocol for stem cell differentiation are the main limitations for achieving suitable cell sources. Moreover, handling of tissue constructs for transplantation and the risk of graft immune rejection are the difficulties for *in vitro* engineered tissue transplantation approaches (Leyendecker et al., 2018[19]; Mazza et al., 2018[28]; Ahmadi et al,. 2018[1]; Zhai et al., 2018[53]; Saheli et al., 2018[39]). It has not been possible to prepare critical physiological signals that are involved in normal tissue regeneration in the body despite recent advances in the field of *in vitro* tissue engineering, the use of various cell combinations, different biomaterials and biomolecules, and dynamic culture conditions (Martinod et al., 2017[26]; Jana et al., 2019[15]). In order to overcome this limitation, some investigations have suggested the use of natural bioreactor “the body” on *in vivo* tissue engineering approaches (Martinod et al., 2017[26]).

In this regard, it is expected that achieving an ideal strategy to create an *in vivo* biomimicking liver construct would need a scaffold that has a suitable microarchitecture and essential biochemical cues, in addition to autologous non-immunogenic cells and biologically active molecules (Mattei et al., 2017[27]).

The goal of this study was the proof-of-concept that is it possible to regenerate a fibrotic liver through *in vivo* tissue engineering using a xenograft of a LEM scaffold and recruitment of autologous cells. Therefore, a xenograft of SDF-1α loaded mouse liver ECM was transplanted in a fibrotic liver rat model along with G-CSF therapy, which reinforced mobilization of rat endogenous bone marrow stem/progenitor cells. Our results demonstrated that this strategy led to increased cell engraftment and differentiation to a hepatic fate in the transplanted LEM, as well as improvements in hepatic functions in the fibrotic liver model.

The transition from cell differentiation and proliferation to formation of a tissue construct is aided by the constant and dynamic interrelationship between cells and the ECM (Nelson and Bissell, 2006[33]). Liver-specific ECM, which is available after organ decellularization could provide a permissive microenvironment to support stem/progenitor cell differentiation to hepatocytes via cell to matrix interactions (Naeem et al., 2019[31]; Mao et al., 2020[25]). Recent findings showed that a specific 3D of the ECM had a significant impact on the ability of stem cells and other cells to regenerate (Badylak, 2007[2]). On the other hand, weak diffusion would not provide oxygen and nutrition to cells of liver constructs and threaten liver cell viability (Zhao et al., 2019[56]). The vascular networks in the native liver ECM could provide a perfusion system and support cell viability (Badylak, 2007[2]; Shimoda et al., 2019[45]). 

Recent advances showed that intrahepatic transplantation of a liver ECM derived scaffold supported migration and survival of hepatic progenitor cells and hepatocytes. Therefore, *in situ* recellularization of this transplanted scaffold could promote hepatic functionality. The results showed better angiogenesis in the liver construct and the major obstacle of construct transplantation, the lack of a perfusion system, was overcome (Saleh et al., 2020[41]).

The aim of some studies was to achieve native LEM with an intact vascular network template by a suitable method for liver decellularization (Mattei et al., 2017[27]). In this study, we used physical and chemical approaches for whole liver decellularization. According to a previous report, our results showed that this method could preserve biochemical characteristics and vascular architecture of the liver in the LEM, which made the scaffold favorable for recellularization and revascularization after transplantation (Jiang et al., 2014[16]). 

Shimoda et al. (2019[45]) transplanted a cell-free decellularized LEM-derived scaffold into a porcine partial hepatectomy model. The acellular scaffold was infiltrated by Alb positive hepatocytes, which implied that the natural liver scaffold retained sufficient biochemical and/or biophysical signals to induce both proliferation and differentiation of nearby liver progenitor cells. These findings suggest that implantation of LEM-derived scaffolds could promote *in situ* liver construct organization after liver resection.

The recruitment of endogenous hematopoietic and mesenchymal stem cells, and endothelial progenitor cells (EPCs) plays a critical role in liver regeneration when hepatocyte compensatory proliferation and liver stem cell activation are insufficient (Ahmadi et al., 2018[1]; Zhai et al., 2018[53]) reported that pharmacological mobilization of stem cells in a partial hepatectomy rat model promoted liver regeneration and improved hepatic functions (Zhai et al., 2018[53]). 

The expression of many chemokines, such as SDF-1α, play critical roles in progression or suppression of liver disease (Liepelt and Tacke, 2016[21]; Saiman et al., 2015[40]). Current studies show a potential protective role by SDF-1α during liver damage and a fundamental role in liver regeneration by activation of liver stem and stellate cells (Liu et al., 2015[22]; Saiman et al., 2015[40]). On the other hand, circulating hematopoietic stem/progenitor cells, MSCs and EPCs contain the CXCR-4 surface marker that has a high affinity to their own specific ligand, SDF-1α (Liepelt and Tacke, 2016[21]). The CXCR-4/SDF-1α pathway could boost migration and homing of such circulating cells in the injured liver and their recruitment in the regeneration process. Liu et al. (2015[22]) reported that increasing SDF-1α is the key factor for promotion of MSCs migration to a fibrotic liver and could attenuate liver damage in a CCl4-induced liver fibrosis mice model.

G-CSF is a hematopoietin that motivates and mobilizes stem/progenitor cells in bone marrow (Chen et al., 2019[4]). G-CSF therapy has been shown to attenuate liver damage and improve liver function through increasing the homing of stem/progenitor cells into liver tissues (Chen et al., 2019[4]). Furthermore, G-CSF could modulate inflammation and provide a suitable context for tissue regeneration (Philips et al., 2019[37]). Chen et al*.* (2019[4]) reported that IP injection of G-CSF increased the efficacy of MSCs therapy in an acute liver failure model. G-CSF ameliorated liver injury by enhancing liver homing and proliferation of the MSCs that were infused through the tail veins of mice. On the other hand, G-CSF inhibited inflammation and liver cell apoptosis via a reduction in oxidative stress and, finally, improved hepatic function.

Nevertheless, in the end-stage liver diseases, collagen fiber synthesis leads to fibrous tissue formation, which restricts cell infiltration and makes liver recovery impossible by pharmacological cell mobilization. Under these situations, tissue engineering approaches should be considered as promising alternatives for liver regeneration (Ahmadi et al., 2018[1]; Hu et al., 2019[14]; Shiha et al., 2020[44]). Cell-free scaffold transplantation and pharmaceutical induction of cell mobilization for *in situ* recellularization of decellularized liver ECM are proposed to be efficient attempts for treatment of a liver fibrosis model. 

Consequently, we investigated attenuation of liver fibrosis by creating bioartificial liver microtissues in the liver microenvironment. We employed cell-free LEM and *in vivo* recellularization optimized by SDF-1α loading on the scaffold and G-CSF treatment. Our results showed a decrease in liver fibrosis after scaffold transplantation and simultaneous treatment with SDF-1α and/or G-CSF. Therefore, there was no difference in the fibrosis stages of the LEM-TxS, LEM-TxG and LEM-TxSG groups compared to the normal group. Damania et al*.* (2018[5]) introduced decellularized liver-derived scaffolds as a potential hepatocyte carrier in bioartificial liver support systems and a regenerative scaffold for *in vivo* tissue engineering. They showed that the scaffolds could recellularize and integrate with native liver tissue to promote liver function in an animal model of liver failure. Moreover, we demonstrated that the simultaneous application of SDF-1α and G-CSF considerably increased cell homing into the transplanted scaffold. Furthermore, differentiation of the engrafted cells into hepatocytes could be significantly induced by simultaneous application of SDF-1α and G-CSF. 

Enhanced engraftment of cells and hepatocytic differentiation can cause improvements in liver function. Our evaluation of liver-specific functionality biomarkers showed that serum levels of ALT and AST in all of the groups that received the scaffold grafts and in the non-transplanted group significantly decreased compared to the fibrotic groups and reached the normal level four weeks after intervention. Reductions in serum ALT and AST levels confirmed recovery of hepatic function after transplantation in the liver fibrosis model (Liao et al., 2020[20]). We observed that Alb secretion was significantly higher in the LEM-TxSG group compared to the fibrotic, non-transplanted, and LEM-Tx groups. Consequently, better liver functionality could be achieved by co-administration of SDF-1α and G-CSF with the LEM transplantation. 

Providing a perfusion system is a critical issue for survival and proliferation of engrafted cells in the scaffolds after transplantation. If this does not happen, a necrotic center in the transplanted construct would be formed (Park et al., 2016[36]). Our decellularization method could protect the vascular architecture of the liver. Therefore, our immunostaining results demonstrated efficient re-endothelialization of the pre-existing vascular network of the scaffold after transplantation. We evaluated the total number of blood vessels four weeks after transplantation and found no differences in the LEM transplanted groups. However, the number of small blood vessels were significantly higher with co-administration of SDF-1α and G-CSF compared to the LEM-Tx and LEM-TxS groups. This might be attributed to mobilization of EPCs and recruitment stimulation by G-CSF therapy and SDF-1α loading on the scaffold (Honold et al., 2006[13]; DeLeve et al. 2016[7]). While a sufficient perfusion system is a limiting factor in *in vitro* tissue engineering approaches, we could provide a hepatic microtissue in the fibrotic liver environment because of maintenance of native vascular microarchitecture of the LEM scaffold; efficient revascularization could support long-term survival of engrafted cells. 

Devalliere et al. (2018[8]) reported that chemical modification of the vascular structure of the LEM-derived scaffold accelerated endothelial progenitor cell recruitment and functional re-endothelialization. DeLeve et al*.* (2016[7]) showed that SDF-1ɑ induced liver sinusoidal endothelial cell recruitment and increased cell attachment and proliferation could lead to lining of the blood vessels by endothelial cells. Fu et al*.* (2016[11]) found that G-CSF, through induction of cell mobilization and recruitment into scaffolds, could provide ideal autologous EPCs to form a functional liver construct. In this regard, our study demonstrated practical re-endothelialization of decellularized liver-derived scaffold by SDF-1ɑ and G-CSF. These data present a promising strategy for a bioengineered liver construct. 

Despite the benefits of bioengineering for human *in vitro* liver construct formation, the need to obtain a scalable and efficient method that provides a liver cell equivalent remains an obstacle. Our results showed that this *in vivo* tissue engineering strategy through mobilization of patient-specific cells would remove this obstacle without the need for immunosuppression upon transplantation. 

## Conclusion

In this work, we attempted to generate regeneration foci in a rat fibrotic liver by transplantation of an exogenous LEM scaffold and induce recruitment of endogenous cells. Our results demonstrated that stem/progenitor cells' mobilization by G-CSF therapy together with induction of chemotaxis in stem cells via SDF-1α loading in the scaffold could improve the regeneration centers, cell differentiation, angiogenesis, and related hepatic function. This work provides a proof-of-concept for feasibility and some efficiency of an *in vivo* tissue engineering approach as a novel therapeutic strategy for treatment of a fibrotic liver. 

## Notes

Hossein Bahadoran and Abbas Piryaei (Department of Biology and Anatomical Sciences, School of Medicine, Shahid Beheshti University of Medical Sciences, Tehran, Iran; Tel: +98 2123872555, E-mail: piryae@sbmu.ac.ir) contributed equally as corresponding author.

## Declaration

### Acknowledgments

This study was financially supported by a grant from the Royan Institute for Stem Cell Biology and Technology (grant no. 91000722) to A.P. The authors wish to express their gratitude to Zahra Azhdari Tafti, Mahshad Dorraj, Mohammad Jafari Atrabi and Payam Taheri for their technical support. 

### Conflict of interest

The authors declare that they have no conflict of interest.

## Supplementary Material

Supplementary information

## References

[1] Ahmadi AR, Chicco M, Wesson RN, Anders RA, Dor FJ, IJzermans JN (2018). Stem cell mobilization is lifesaving in a large animal preclinical model of acute liver failure. Ann Surg.

[2] Badylak SF (2007). The extracellular matrix as a biologic scaffold material. Biomaterials.

[3] Bukenya F, Nerissa C, Serres S, Pardon M-C, Bai L (2020). An automated method for segmentation and quantification of blood vessels in histology images. Microvasc Res.

[4] Chen H, Tang S, Liao J, Liu M, Lin Y (2019). Therapeutic effect of human umbilical cord blood mesenchymal stem cells combined with G-CSF on rats with acute liver failure. BBRC.

[5] Damania A, Kumar A, Teotia AK, Kimura H, Kamihira M, Ijima H (2018). Decellularized liver matrix-modified cryogel scaffolds as potential hepatocyte carriers in bioartificial liver support systems and implantable liver constructs. ACS Aapp Mater Inter.

[6] Danilova IG, Yushkov BG, Kazakova IA, Belousova AV, Minin AS, Abidov MT (2019). Recruitment of macrophages and bone marrow stem cells to regenerating liver promoted by sodium phthalhydrazide in mice. Biomed Pharmacother.

[7] DeLeve LD, Wang X, Wang L (2016). VEGF-sdf1 recruitment of CXCR7+ bone marrow progenitors of liver sinusoidal endothelial cells promotes rat liver regeneration. Am J Physiol Gastrointest Liver Physiol.

[8] Devalliere J, Chen Y, Dooley K, Yarmush ML, Uygun BE (2018). Improving functional re-endothelialization of acellular liver scaffold using REDV cell-binding domain. Acta Biomat.

[9] Eom YW, Shim KY, Baik SK (2015). Mesenchymal stem cell therapy for liver fibrosis. Korean J Intern Med.

[10] Forbes SJ, Gupta S, Dhawan A (2015). Cell therapy for liver disease: from liver transplantation to cell factory. J Hepatology.

[11] Fu W-L, Xiang Z, Huang F-G, Cen S-Q, Zhong G, Liu M (2016). Combination of granulocyte colony-stimulating factor and CXCR4 antagonist AMD3100 for effective harvest of endothelial progenitor cells from peripheral blood and in vitro formation of primitive endothelial networks. Cell Tissue Bank.

[12] Fu X, Liu G, Halim A, Ju Y, Luo Q, Song G (2019). Mesenchymal stem cell migration and tissue repair. Cells.

[13] Honold J, Lehmann R, Heeschen C, Walter DH, Assmus B, Sasaki K-I (2006). Effects of granulocyte colony stimulating factor on functional activities of endothelial progenitor cells in patients with chronic ischemic heart disease. Arterioscler Thromb Vasc Biol.

[14] Hu C, Zhao L, Duan J, Li L (2019). Strategies to improve the efficiency of mesenchymal stem cell transplantation for reversal of liver fibrosis. J Cell Mol Med.

[15] Jana S, Franchi F, Lerman A (2019). Trilayered tissue structure with leaflet-like orientations developed through in vivo tissue engineering. Biomed Mater.

[16] Jiang W-C, Cheng Y-H, Yen M-H, Chang Y, Yang VW, Lee OK (2014). Cryo-chemical decellularization of the whole liver for mesenchymal stem cells-based functional hepatic tissue engineering. Biomaterials.

[17] Lee H, Han W, Kim H, Ha D-H, Jang J, Kim BS (2017). Development of liver decellularized extracellular matrix bioink for three-dimensional cell printing-based liver tissue engineering. Biomacromolecules.

[18] Lee JW, Choi Y-J, Yong W-J, Pati F, Shim J-H, Kang KS (2016). Development of a 3D cell printed construct considering angiogenesis for liver tissue engineering. Biofabrication.

[19] Leyendecker Junior A, Gomes Pinheiro CC, Lazzaretti Fernandes T, Franco Bueno D (2018). The use of human dental pulp stem cells for in vivo bone tissue engineering: a systematic review. J Tissue Eng.

[20] Liao N, Shi Y, Wang Y, Liao F, Zhao B, Zheng Y (2020). Antioxidant preconditioning improves therapeutic outcomes of adipose tissue-derived mesenchymal stem cells through enhancing intrahepatic engraftment efficiency in a mouse liver fibrosis model. Stem Cell Res Ther.

[21] Liepelt A, Tacke F (2016). Stromal cell-derived factor-1 (SDF-1) as a target in liver diseases. Am J Physiol Gastrointest Liver Physiol.

[22] Liu Y, Yang X, Jing Y, Zhang S, Zong C, Jiang J (2015). Contribution and mobilization of mesenchymal stem cells in a mouse model of carbon tetrachloride-induced liver fibrosis. Sci Rep.

[23] Lotfinia M, Kadivar M, Piryaei A, Pournasr B, Sardari S, Sodeifi N (2016). Effect of secreted molecules of human embryonic stem cell-derived mesenchymal stem cells on acute hepatic failure model. Stem Cells Dev.

[24] Ma X, Qu X, Zhu W, Li Y-S, Yuan S, Zhang H (2016). Deterministically patterned biomimetic human iPSC-derived hepatic model via rapid 3D bioprinting. Proc Natl Acad Sci U S A.

[25] Mao Q, Wang Y, Li Y, Juengpanich S, Li W, Chen M (2020). Fabrication of liver microtissue with liver decellularized extracellular matrix (dECM) bioink by digital light processing (DLP) bioprinting. Mater Sci Eng C Mater Biol Appl.

[26] Martinod E, Paquet J, Dutau H, Radu DM, Bensidhoum M, Abad S (2017). In vivo tissue engineering of human airways. Ann Thorac Surg.

[27] Mattei G, Magliaro C, Pirone A, Ahluwalia A (2017). Decellularized human liver is too heterogeneous for designing a generic extracellular matrix mimic hepatic scaffold. Artif Organs.

[28] Mazza G, Al‐Akkad W, Rombouts K, Pinzani M (2018). Liver tissue engineering: From implantable tissue to whole organ engineering. Hepatol Commun.

[29] Mazza G, Rombouts K, Hall AR, Urbani L, Luong TV, Al-Akkad W (2015). Decellularized human liver as a natural 3D-scaffold for liver bioengineering and transplantation. Sci Rep.

[30] McCullen SD, Chow AG, Stevens MM (2011). In vivo tissue engineering of musculoskeletal tissues. Curr Opin Biotechnol.

[31] Naeem EM, Sajad D, Talaei‐Khozani T, Khajeh S, Azarpira N, Alaei S (2019). Decellularized liver transplant could be recellularized in rat partial hepatectomy model. J Biomed Mater Res A.

[32] Namiri M, Kazemi Ashtiani M, Abbasalizadeh S, Mazidi Z, Mahmoudi E, Nikeghbalian S (2018). Improving the biological function of decellularized heart valves through integration of protein tethering and three-dimensional cell seeding in a bioreactor. J Tissue Eng Regen Med.

[33] Nelson CM, Bissell MJ (2006). Of extracellular matrix, scaffolds, and signaling: tissue architecture regulates development, homeostasis, and cancer. Annu Rev Cell Dev Biol.

[34] Nobakht Lahrood F, Saheli M, Farzaneh Z, Taheri P, Dorraj M, Baharvand H (2020). Generation of transplantable three-dimensional hepatic-patch to improve the functionality of hepatic cells in vitro and in vivo. Stem Cells Dev.

[35] Oldhafer F, Bock M, Falk CS, Vondran FW (2016). Immunological aspects of liver cell transplantation. World J Transplant.

[36] Park K-M, Hussein KH, Hong S-H, Ahn C, Yang S-R, Park S-M (2016). Decellularized liver extracellular matrix as promising tools for transplantable bioengineered liver promotes hepatic lineage commitments of induced pluripotent stem cells. Tissue Eng Part A.

[37] Philips CA, Augustine P, Ahamed R, Rajesh S, George T, Valiathan GC (2019). Role of granulocyte colony-stimulating factor therapy in cirrhosis,‘inside any deep asking is the answering’. J Clin Transl Hepatol.

[38] Rosai A (2011). Rosai and Ackerman's surgical pathology.

[39] Saheli M, Sepantafar M, Pournasr B, Farzaneh Z, Vosough M, Piryaei A (2018). Three‐dimensional liver‐derived extracellular matrix hydrogel promotes liver organoids function. J Cell Biochem.

[40] Saiman Y, Jiao J, Fiel MI, Friedman SL, Aloman C, Bansal MB (2015). Inhibition of the CXCL 12/CXCR 4 chemokine axis with AMD 3100, a CXCR 4 small molecule inhibitor, worsens murine hepatic injury. Hepatol Res.

[41] Saleh T, Ahmed E, Yu L, Song SH, Park KM, Kwak HH (2020). Conjugating homogenized liver‐extracellular matrix into decellularized hepatic scaffold for liver tissue engineering. J Biomed Mater Res A.

[42] Sauer IM, Raschzok N, Neuhaus P (2014). The artificial liver, in vivo tissue engineering, and organ printing. Textbook of organ transplantation.

[43] Sellaro TL, Ranade A, Faulk DM, McCabe GP, Dorko K, Badylak SF (2010). Maintenance of human hepatocyte function in vitro by liver-derived extracellular matrix gels. Tissue Eng Part A.

[44] Shiha G, Nabil A, Lotfy A, Soliman R, Hassan AA, Ali IS (2020). Antifibrotic effect of combination of nilotinib and stem cell-conditioned media on CCl4-induced liver fibrosis. Stem Cells Int.

[45] Shimoda H, Yagi H, Higashi H, Tajima K, Kuroda K, Abe Y (2019). Decellularized liver scaffolds promote liver regeneration after partial hepatectomy. Sci Rep.

[46] Tadakuma K, Tanaka N, Haraguchi Y, Higashimori M, Kaneko M, Shimizu T (2013). A device for the rapid transfer/transplantation of living cell sheets with the absence of cell damage. Biomaterials.

[47] Uygun BE, Soto-Gutierrez A, Yagi H, Izamis M-L, Guzzardi MA, Shulman C (2010). Organ reengineering through development of a transplantable recellularized liver graft using decellularized liver matrix. Nat Med.

[48] Vishwakarma SK, Bardia A, Lakkireddy C, Raju N, Paspala SAB, Habeeb MA (2019). Intraperitoneal transplantation of bioengineered humanized liver grafts supports failing liver in acute condition. Mater Sci Eng C Mater Biol Appl.

[49] Wallace MC, Hamesch K, Lunova M, Kim Y, Weiskirchen R, Strnad P (2015). Standard operating procedures in experimental liver research: thioacetamide model in mice and rats. Lab Anim.

[50] Wan Z, Zhang XG, Liu ZW, Lv Y (2013). Therapeutic liver repopulation for metabolic liver diseases: Advances from bench to bedside. Hepatol Res.

[51] Wesson RN, Cameron AM (2011). Stem cells in acute liver failure. Adv Surg.

[52] Yang J-G, He X-F, Huang B, Zhang H-A, He Y-K (2018). Rule of changes in serum GGT levels and GGT/ALT and AST/ALT ratios in primary hepatic carcinoma patients with different AFP levels. Cancer Biomark.

[53] Zhai R, Wang Y, Qi L, Williams GM, Gao B, Song G (2018). Pharmacological mobilization of endogenous bone marrow stem cells promotes liver regeneration after extensive liver resection in rats. Sci Rep.

[54] Zhang J, Zhao X, Liang L, Li J, Demirci U, Wang S (2018). A decade of progress in liver regenerative medicine. Biomaterials.

[55] Zhang Y, He Y, Bharadwaj S, Hammam N, Carnagey K, Myers R (2009). Tissue-specific extracellular matrix coatings for the promotion of cell proliferation and maintenance of cell phenotype. Biomaterials.

[56] Zhao Y, Xu B, Liang W, Ding Y, Li J, Zhang Y (2019). Multisite injection of bioengineered hepatic units from collagen hydrogel and neonatal liver cells in parenchyma improves liver cirrhosis. Tissue Eng Part A.

