# Supplementary material for: Transplantation of SDF-1α-loaded liver extracellular matrix repopulated with autologous cells attenuated liver fibrosis in a rat model
Source: EXCLI J. 2022 Apr 22;21:704–21. doi: 10.17179/excli2022-4761 (PMC9203988; doi:10.17179/excli2022-4761)
Supplement: Supplementary information [file EXCLI-21-704-s-001.pdf]

EXCLI Journal 2022;21:704-721 – ISSN 1611-2156
Received: February 11, 2022, accepted: April 20, 2022, published: April 22, 2022

Supplementary information to:

Original article:

TRANSPLANTATION OF SDF-1α-LOADED LIVER EXTRACELLULAR MATRIX REPOPULATED WITH AUTOLOGOUS CELLS ATTENUATED LIVER FIBROSIS IN A RAT MODEL

Mostafa Najar-Asl[1,2], Hossein Bahadoran[2*], Mohammad-Hossein Asadi[2], Mona Saheli[3], Mohammad-Hassan Asghari[4], Niloofar Sodeifi[5], Mohammad Kazemi Ashtiani[6], Massoud Vosough[7], Hossein Baharvand[1,8], Abbas Piryaei[1,9,10]*

1 Department of Stem Cells and Developmental Biology, Cell Science Research Center, Royan Institute for Stem Cell Biology and Technology, ACECR, Tehran, Iran
2 Department of Anatomical Sciences, School of Medical Sciences, Baqiyatallah University, Tehran, Iran
3 Department of Anatomical Sciences, Afzalipour Faculty of Medicine, Kerman University of Medical Sciences, Kerman, Iran
4 Animal Core Facility, Reproductive Biomedicine Research Center, Royan Institute for Biotechnology, ACECR, Tehran, Iran
5 Department of Andrology, Reproductive Biomedicine Research Center, Royan Institute for Reproductive Biomedicine, ACECR, Tehran, Iran
6 Department of Cell Engineering, Cell Science Research Center, Royan Institute for Stem Cell Biology and Technology, ACECR, Tehran, Iran
7 Department of Regenerative Medicine, Cell Science Research Center, Royan Institute for Stem Cell Biology and Technology, ACECR, Tehran, Iran
8 Department of Developmental Biology, School of Basic Sciences and Advanced Technologies in Biology, University of Science and Culture, Tehran, Iran
9 Department of Biology and Anatomical Sciences, School of Medicine, Shahid Beheshti University of Medical Sciences, Tehran, Iran
10 Department of Tissue Engineering and Applied Cell Sciences, School of Advanced Technology in Medicine, Shahid Beheshti University of Medical Sciences, Tehran, Iran

* **Corresponding authors:** Hossein Bahadoran, Department of Anatomical Sciences, School of Medical Sciences, Baqiyatallah University, Tehran, Iran. Tel: +98 9124276200; E-mail: bahadoran.h@bmsu.ac.ir
Abbas Piryaei, Department of Biology and Anatomical Sciences, School of Medicine, Shahid Beheshti University of Medical Sciences, Tehran, Iran. Tel: +98 2123872555; E-mail: piryae@sbmu.ac.ir

https://dx.doi.org/10.17179/excli2022-4761

This is an Open Access article distributed under the terms of the Creative Commons Attribution License (http://creativecommons.org/licenses/by/4.0/).

EXCLI Journal 2022;21:704-721 – ISSN 1611-2156
Received: February 11, 2022, accepted: April 20, 2022, published: April 22, 2022

Supplementary Table 1: The description of fibrosis stages and necrosis grades in liver

Fibrosis stage		Description
0		Normal liver (No fibrosis)
1		Fibrous expansion of some portal areas, with or without short fibrous septa
2		Fibrous expansion of most portal areas, with or without short fibrous septa
3		Fibrous expansion of most portal areas, with occasional portal-portal (P-P) bridging
4		Fibrous expansion of portal areas, with marked P-P bridging as well as some portal-central (P-C) bridging
5		Marked P-P and/or P-C bridging, with occasional nodules (incomplete cirrhosis)
6		Cirrhosis, probable or definite
**Necrosis grades***		**Description**
Piecemeal necrosis	0	Absent: no visible hepatocyte necrosis
	1	Focal necrosis: few portal areas
	2	Mild/Moderate necrosis: most portal areas
	3	Moderate necrosis: continuous around <50 % of tracts or septa
	4	Severe necrosis: continuous around >50 % of tracts or septa
Confluent necrosis	0	Absent: no visible hepatocyte necrosis
	1	Focal confluent necrosis
	2	Centrolobular necrosis in some areas
	3	Centrolobular necrosis in most areas
	4	Centrolobular necrosis + occasional P-C bridging
	5	Centrolobular necrosis + multiple P-C bridging
	6	Pan-lobular or multi-lobular necrosis
Focal lytic necrosis apop-tosis and focal inflammation	0	Absent: no visible hepatocyte necrosis
	1	One focus or less per 10x objective
	2	One to four foci per 10x objective
	3	Five to 10 foci per 10x objective
	4	More than ten foci per 10x objective
Portal inflammation	0	Absent: none portal inflammation
	1	Mild: some or all portal areas
	2	Moderate: some or all portal areas
	3	Moderate/marked: all portal areas
	4	Marked: all portal areas

*Maximum possible score for grading is 18.
